# Postsurgical motor function and processing speed as predictors of quality of life in patients with chronic-phase glioblastoma

**DOI:** 10.1007/s00701-024-06245-1

**Published:** 2024-08-31

**Authors:** Riho Nakajima, Masashi Kinoshita, Hirokazu Okita, Mitsutoshi Nakada

**Affiliations:** 1 Department of Occupational Therapy, Faculty of Health Science, Institute of Medical, Pharmaceutical and Health Sciences, Kanazawa University, Kanazawa, Japan; 2 Department of Neurosurgery, Faculty of Medicine, Institute of Medical, Pharmaceutical and Health Sciences, Kanazawa University, Kanazawa, Japan; 3 Department of Physical Medicine and Rehabilitation, Kanazawa University Hospital, Kanazawa, Japan; 4 Department of Neurosurgery, Division of Neuroscience, Graduate School of Medical Science, Kanazawa University, 13-1 Takara-machi, Kanazawa, 920-8641 Ishikawa Japan

**Keywords:** Glioblastoma, Quality of life, Brain function, Motor function, Processing speed

## Abstract

**Purpose:**

Patients with glioblastomas (GBMs) have poor prognosis despite various treatments; therefore, attention should be paid to maintaining the quality of survival. Neurocognitive deficits can affect the quality of life (QOL) in patients with GBM. Most studies concerning QOL and neurocognitive functions have demonstrated a relationship between QOL and self-reported neurocognitive decline, although this method does not accurately reflect damaged functional domains. Therefore, this study aimed to clarify the neurocognitive functions that influence the QOL in patients with GBMs using an objective assessment of neurocognitive functions.

**Methods:**

Data from 40 patients newly diagnosed with GBMs were analyzed. All patients completed the assessment of QOL and various neurological and neurocognitive functions including general cognitive function, processing speed, attention, memory, emotion recognition, social cognition, visuospatial cognition, verbal fluency, language, motor function, sensation, and visual field at 6 months postoperatively. QOL was assessed using the 36-Item Short Form Survey (SF-36). In the SF-36, the physical, mental, and role and social component summary (PCS, MCS, and RCS, respectively) scores were calculated. Multiple logistic regression analyses and chi-square tests were used to evaluate the association between SF-36 scores and neurocognitive functions.

**Results:**

The MCS was maintained, while the PCS and RCS scores were significantly lower in patients with GBMs than in healthy controls (*p* = 0.0040 and *p* < 0.0001, respectively). Among several neurocognitive functions, motor function and processing speed were significantly correlated with PCS and RCS scores, respectively (*p* = 0.0048 and *p* = 0.030, respectively). Patients who maintained their RCS or PCS scores had a higher probability of preserving motor function or processing speed than those with low RCS or PCS scores (*p* = 0.0026).

**Conclusions:**

Motor function and processing speed may be predictors of QOL in patients with GBMs.

**Supplementary Information:**

The online version contains supplementary material available at 10.1007/s00701-024-06245-1.

## Introduction

Quality of life (QOL) is “a conscious cognitive judgment of satisfaction with one’s life” [24]. Patients with glioblastoma (GBM) have a poor prognosis, with an average survival of less than 2 years despite various treatment attempts [38]. Several studies have investigated QOL in patients with GBM [4, 6, 36]. This is because GBM is highly lethal; therefore, emphasis has been placed on prolonging overall survival and maintaining QOL. Traditionally, clinicians have focused on providing surgical treatment for tumors and prolonging the progression-free or overall survival. Recently, emphasis has been placed on considering patients’ QOL and emotional well-being when implementing treatment strategies [54].

The factors affecting QOL in patients with GBM include the Karnofsky Performance Status (KPS) score, age, sex, tumor location, depression, treatment, and brain function [4, 6]. With regard to brain function, motor weakness may result in poor QOL [23, 31, 36], and priority has been placed on maintaining motor function while treating GBM. Furthermore, some studies have reported the influence of neurocognitive decline, such as general cognitive deficits and aphasia, on QOL in patients with GBM [4, 16]. However, the relationship between QOL and neurocognitive function has often been evaluated using patient-reported questionnaires [16, 20, 36] rather than objective measures. The self-reported neurocognitive decline is useful for a comprehensive estimation of patients’ subjective neurocognitive function [39], but it does not necessarily correlate with neurocognitive test results and accurately reflects neurocognitive damage [33, 40]. Taken together, only a few studies have investigated the relationship between neurocognitive function based on objective measures and QOL in patients with GBM. We previously reported that postoperative decline in executive function, language, and motor function influences QOL in patients with lower-grade glioma based on QOL assessments and objective neurocognitive evaluation [31]. Patients with lower-grade glioma and those with GBM exhibit differences in QOL and neurocognitive function, underscoring the necessity to evaluate these individuals separately [16, 30]. Understanding the specific brain functions that affect the QOL of patients with GBM is crucial for developing surgical strategies and care plans for postoperative treatment.

In clinical practice, the term functional outcome, such as independence level, is sometimes equated with QOL. However, functional outcomes do not necessarily conform with QOL [31, 46, 53]. QOL has been assessed using quantitative measures since the 1970s [5, 52]. The two types of QOL assessments commonly used are disease-specific and generic measures for the general population. The 30-item European Organization for Research and Treatment of Cancer Core Quality of Life Questionnaire is the most widely used assessment of health-related QOL worldwide and has been validated for reliability and validity in patients with cancer, including those with brain tumors [1]. In contrast, the 36-Item Short Form Survey (SF-36) was developed as a common measure to compare the functional status and well-being of symptomatic patients with those of the general population. Moreover, it quantitatively analyzes and subjectively evaluates health status from the patient’s perspective [45, 50]. The SF-36, a generic measure, was applied in this study to quantitatively analyze the association between subjective QOL and neurocognitive function in patients with GBM. The self-report questionnaire comprised 36 items. The raw scores of all 36 items were categorized into three summary component scores: physical component summary (PCS), mental component summary (MCS), and role and social component summary (RCS) scores [45]. The SF-36 has been used in previous studies to assess QOL in patients with GBM, and its reliability and validity have been verified [7, 8].

We then investigated which brain functions were related to QOL in patients with GBM. This study aimed to accurately identify the neurological and neurocognitive functions that influence QOL in patients with GBM at 6 months after surgery, or the chronic phase, by conducting objective assessments of several functional domains and subjective assessments of QOL.

## Methods and materials

### Participants

**Fig. 1:**
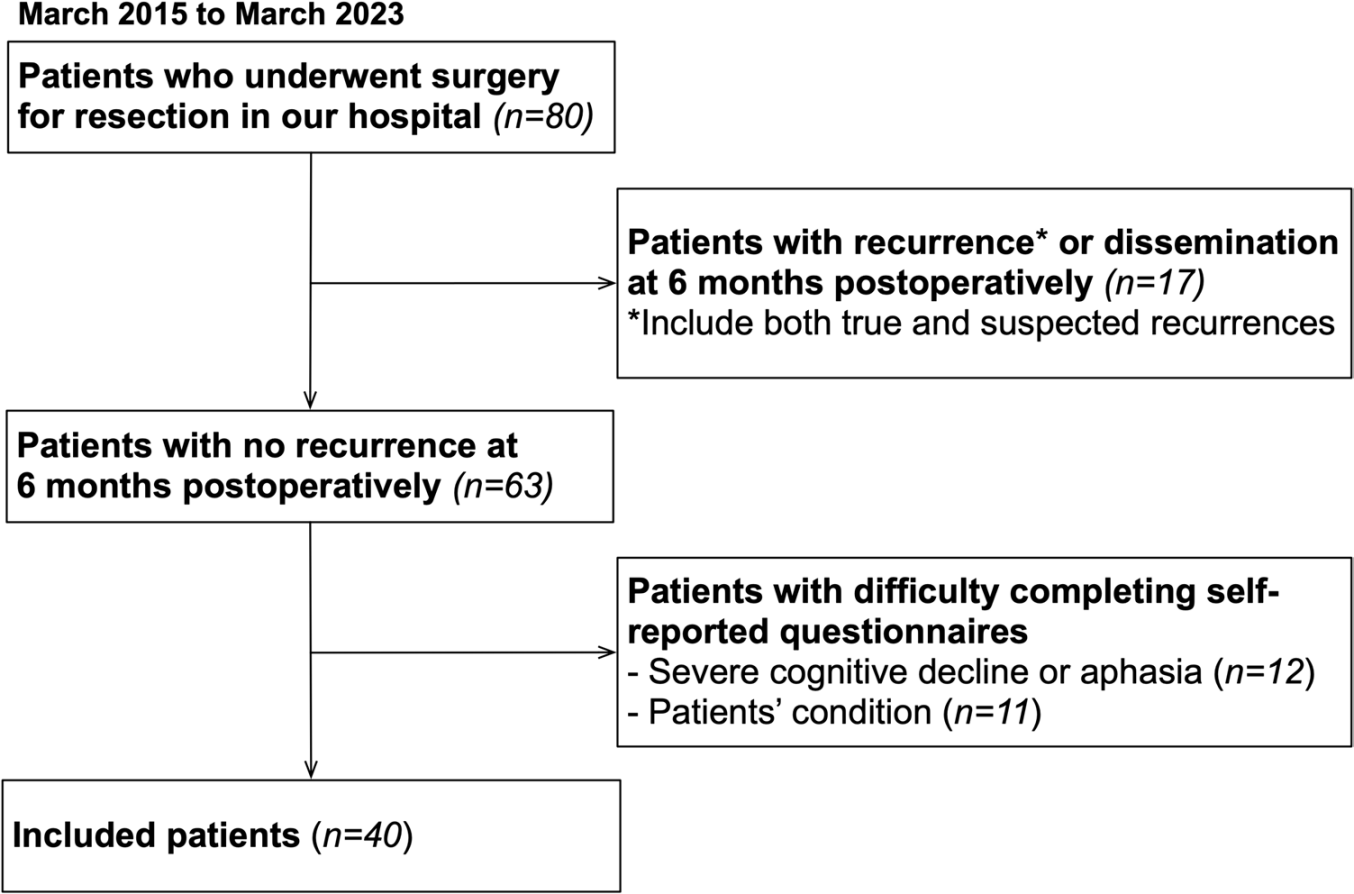
Flow chart of the participant inclusion process

Data from 80 patients with newly diagnosed GBMs and wild-type isocitrate dehydrogenase who underwent surgical resection at Kanazawa University Hospital between March 2015 and March 2023 were reviewed. Patients who experienced tumor recurrence, including true and suspected recurrence, or dissemination at 6 months after surgery (*n* = 17) were excluded due to the known association between tumor progression and decreased QOL [36, 46]. Moreover, patients who were unable to complete the self-reported questionnaire during the chronic phase (*n* = 12) because of severe neurocognitive deficits or language deficits were excluded (Fig. 1). The neurocognitive assessments in this study were performed as part of the standard care in clinical practice. However, some patients with GBMs were unable to complete these assessments, including the neurological/neurocognitive and QOL assessments, on account of fatigue or reduced motivation, resulting in their exclusion from the analysis (*n* = 11). Consequently, 40 patients were included in this study. The patient details are summarized in Table 1. The patients were judged as demonstrating a “return to social life” if they had nearly resumed their previous social activities. The extent of resection was determined based on the volume of gadolinium-enhanced lesions observed on T1-weighted images. This study was conducted in accordance with the Declaration of Helsinki and the guidelines of the Institutional Review Board and approved by the Medical Ethics Committee of Kanazawa University (approval numbers: 1731 and 1797). Written informed consent was obtained from all patients.

**Table 1:** Demographic and clinical characteristic of participants

Characteristics	Value
Age	
Mean ± SD	57.7 ± 12.9
Range	18 to 71
Sex	
Male	18 (45%)
Female	22 (55%)
Laterality	
Left	16 (40%)
Right	24 (60%)
Tumor location	
Frontal	14 (35%)
Parietal	8 (20%)
Temporal	13 (33%)
Occipital	2 (5%)
Insula	3 (7%)
Pre-op tumor volume (cm^3^)	28.3 ± 26.9
Extent of resection (%)	99.1 ± 3.1
MGMT promoter methylation	
Methylated	24 (60%)
Unmethylated	16 (40%)
KPS in post-operative six months	
Mean ± SD	86.3 ± 11.0
Range	60 to 100
Median	90
Return to social life	
Yes	14 (35%)
No	26 (65%)
Postoperative treatment	
Temozolomide	40 (100%)
Irradiation, 60 Gy	33 (82%)
Irradiation, 40 Gy	7 (18%)

*KPS* Karnofsky performance status, *SD* standard deviation

### Outcome measure and definitions

The primary outcome measure was the SF-36 QOL score. All patients completed the SF-36 at 6 months postoperatively. Among the raw component scores of the SF-36, the scores of the eight subcomponents and three summary components were calculated. The summary component scores were used as the outcome measures. The summary component score includes the PCS scores, indicating physical health; the RCS scores, indicating the physical and mental health roles in professional or household activities and participation in social life; and the MCS scores, indicating emotional health performance, such as mental health and vitality [45]. Standard values from 2,279 healthy individuals were provided for these scores [15], with a mean of 50 and a standard deviation (SD) of 10. A “low-level” QOL was defined as a summary component score of less than 40 (mean – 1SD).

The secondary outcome measures included the neurological and neurocognitive test scores. The following items were used to assess neurocognitive function: the Mini-Mental State Examination (MMSE) [13] for general cognitive function; letter cancellation test (time required and the number of errors) for processing speed and attention, respectively [11, 26]; digit span (forward and backward) for memory [11]; expression recognition test for adults for emotion recognition [25]; picture arrangement test for social cognition [29, 51]; line bisection test for visuospatial cognition [21]; verbal fluency test of the phonemic word (e.g. “ka”, the Japanese kana character) and semantic word (e.g. the category with an animal) for verbal fluency [26]; and objective naming test of high-frequency words that a part of Supplementary tests for Standard language test of aphasia [10]. Neurological functions were assessed to determine the presence of motor weakness (paresis), sensory deficits, and visual field deficits. Motor function was assessed using the manual muscle test and the Brunnstrom recovery stage index, which is the commonly used index for evaluating the severity of paresis [37]. A manual muscle test result of ≤ 4 due to paresis was defined as a “deficit.” Both superficial sensations (sense of touch, temperature, pain, and pressure) and deep sensations (sense of muscle and tendon movement) were evaluated. The visual field deficits were defined as greater than quadrantanopia, according to the results of an ophthalmologic visual field assessment. All assessments, including neurological and neurocognitive assessments and SF-36, were performed 6 months postoperatively. All neurocognitive assessments and SF-36 questionnaires were administered by a well-trained occupational therapist (R. N.).

### Neurosurgical procedure

Surgery was performed with the goal of maximal tumor resection and minimal risk of permanent postoperative deficit. To fulfill the oncological priorities in patients with GBM, we resected the central part of the tumor, such as the enhanced areas, in all patients. We routinely used 5-aminolevulinic acid fluorescence guidance to maximally increase the extent of resection. All patients underwent preoperative imaging including diffusion tensor imaging tractography. Continuous intraoperative monitoring via transcortical motor evoked potentials or intraoperative awake stimulation mapping were used to preserve neurological and neurocognitive functions.

### Statistical analysis

The neurocognitive function scores were converted into age-adjusted Z-scores. Converting the Z-score is a common procedure when some test scores are used in research [12]. A Z-score of < 2.0 indicated an “impairment” [27]. For the SF-36, the summary component scores were calculated and standardized based on the national standard value, which was adjusted to a mean of 50 and an SD of 10. A one-sample t-test was performed to compare the summary component scores of the patients and healthy individuals. To identify the functional factors related to the summary component score, we used Pearson’s correlation analysis, t-test, and multiple regression analyses using stepwise methods. The chi-square test was used to examine the relationship between functional factors and low-level QOL. Statistical significance was set at a p-value of < 0.05. We also performed Principal component analysis (PCA) for all neurocognitive functions. In PCA, factors with an eigenvalue greater than one were extracted. Then we analyzed relationships between principal component loading identified in the PCA and SF-36 score. All data were analyzed using the JMP Pro statistical analysis software version 16.2.0 (SAS Institute Japan Inc., Tokyo, Japan) and SPSS statistics 29.0.1.0. (IBM Japan Ltd., Tokyo, Japan). We estimated that a sample size of at least 35 patients would provide sufficient power to perform statistical analyses. The sample size was calculated based on a power of 0.8 and a significance level (α) of 0.05 using G*Power 3.1.9.6.

## Results

### Demographic and clinical characteristics

**Fig. 2:**
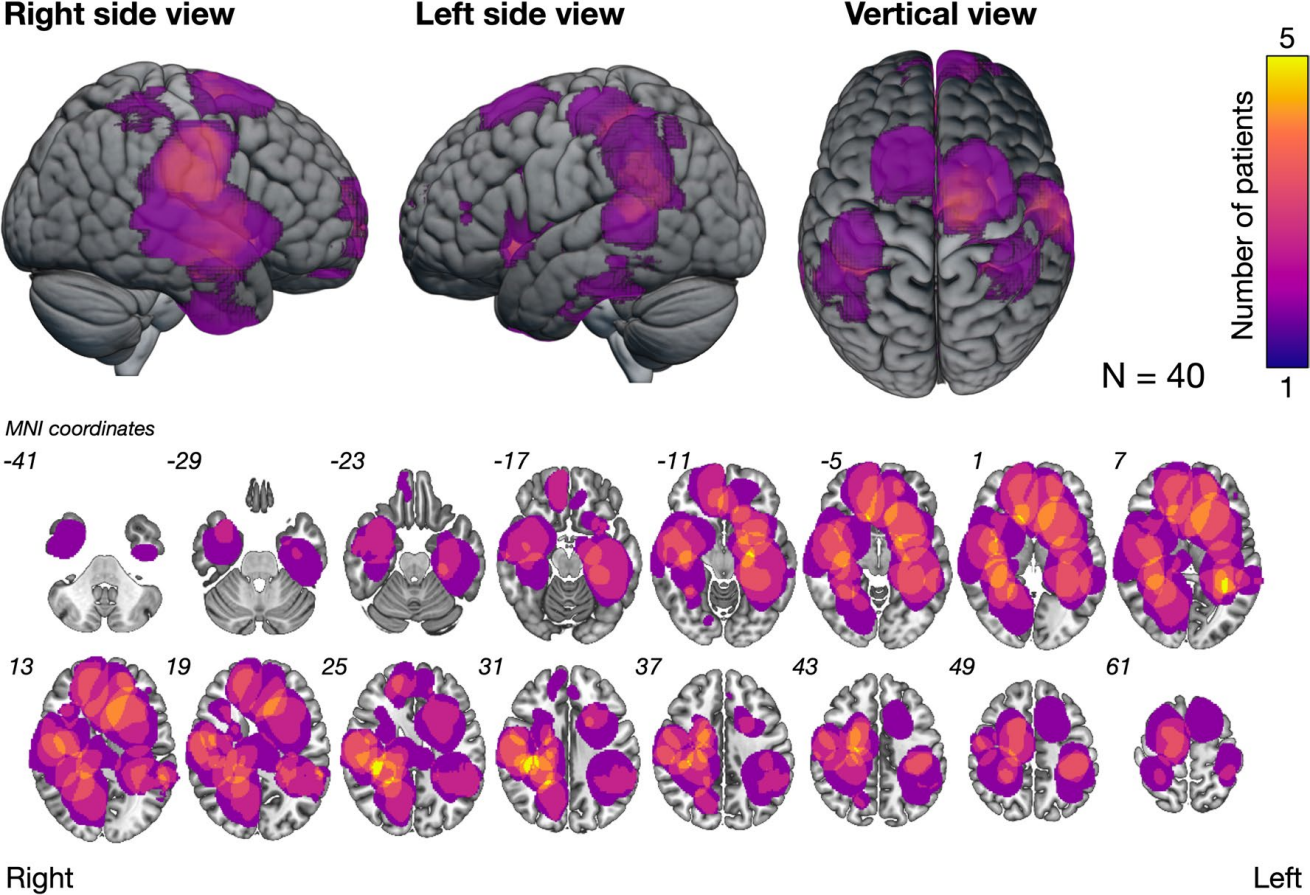
Maps of tumor overlap across all patients (*N* = 40). Yellow regions indicate the most significant overlap in our case group (*N* = 5). Numbers at the upper left of the slices indicate the coordinates of the MNI template MNI, Montreal Neurological Institute

**Fig. 3:**
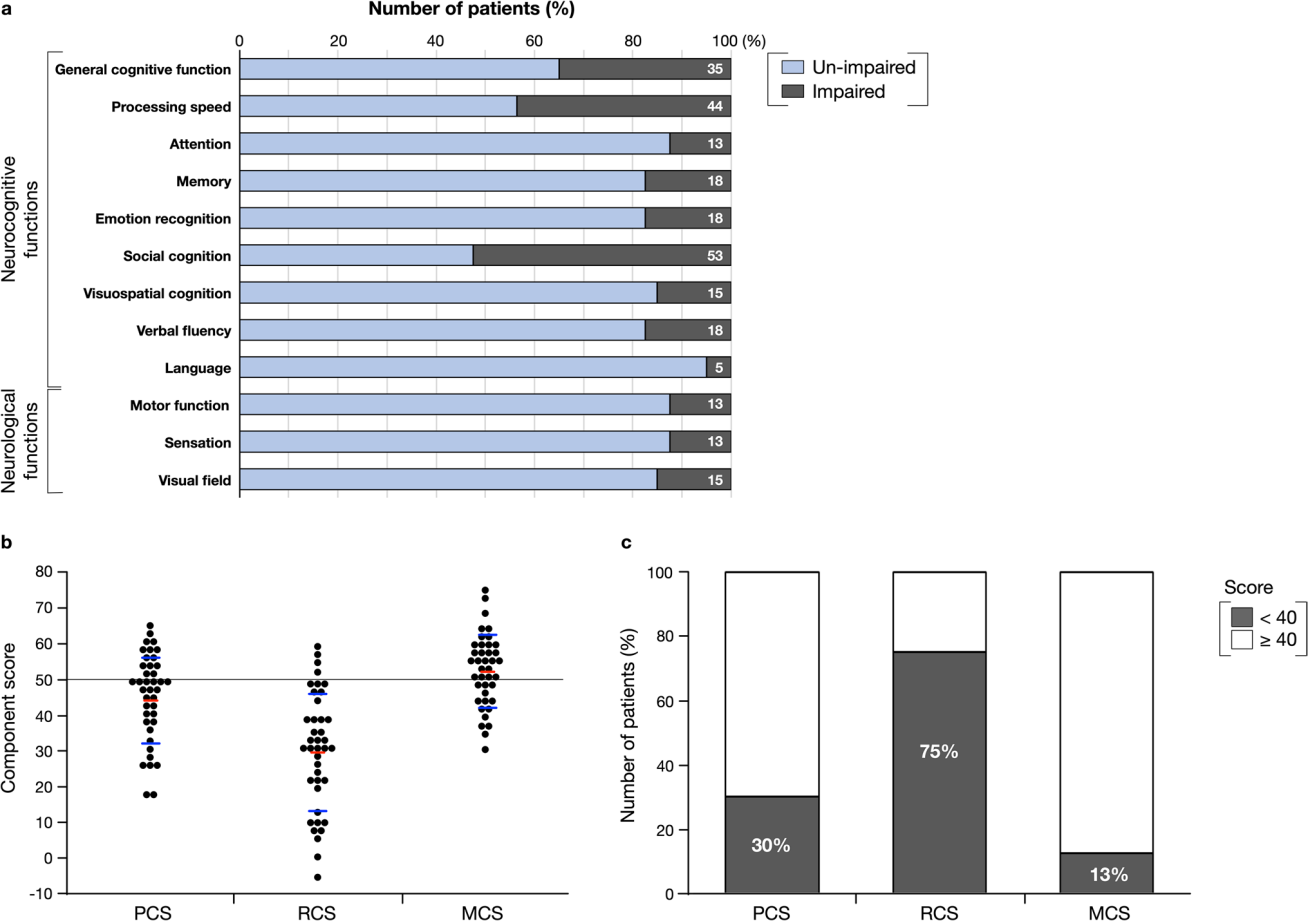
**a** The upper column shows the neurocognitive functions and neurological functions at 6 months postoperatively. Light blue, not impaired; gray, impaired. **b** The lower columns show the results of three summary component scores. Using one-sample Wilcoxon tests, the mental component summary (MCS) scores showed normal distribution, while the physical component summary (PCS) and the role and social component summary (RCS) scores were significantly lower than the normal standard value (mean = 50, standard deviation = 10). Red line: mean; blue line: standard deviation. **c** The PCS and RCS scores were relatively low (score < 40) in 30.0% and 75.0% of patients, respectively. Only 12.5% patients showed a reduction in MCS scores. Dark color: score < 40; light color: score ≥ 40

The demographic and clinical characteristics of the patients are summarized in Table 1. Tumor locations of all patients are shown in Fig. 2. All patients received temozolomide and irradiation following the maximal resection of enhanced lesions: seven older patients received 40 Gy [35], while other patients received 60 Gy. All patients were still on temozolomide maintenance therapy at 6 months postoperatively. Fourteen patients (35%) resumed their previous social activities at 6 months postoperatively, while 26 patients (65%) did not. Although 35% of the patients showed mild cognitive decline (Fig. 3a), all except two patients with a KPS score of 60 did not exhibit communication problems and were able to perform daily activities independently. Of the two patients with a KPS score of 60, one had paresis due to motor area lesions, while the other had memory impairment due to hippocampal lesions. Both of these patients demonstrated normal communication skills but required assistance in performing daily activities. The mean MMSE score at 6 months postoperatively was 27 ± 3.3. Among various neurocognitive functions, social cognition was the most frequently impaired 53%, followed by processing speed 44% (Fig. 3a). With regard to neurological function, 13% of patients experienced paresis or sensory deficits, while 15% had visual field deficits.

### SF-36

In the summary component score, the PCS (mean: 44.1 ± 12.0, range: 17.5 to 63.5, *p* = 0.0040) and RCS scores (mean: 29.6 ± 16.4, range: −5.6 to 58.9, *p* < 0.0001) of the patients were significantly lower than the normal standard value (mean: 50.0, SD: 10.0) (Fig. 3b). The PCS and RCS scores decreased in 30% and 75% of patients, respectively (Fig. 3c). However, the MCS score was largely comparable between patients and healthy individuals (mean: 52.3 ± 10.1, range: 30.2–74.9, *p* = 0.14). Subsequently, the PCS and RCS scores were analyzed. No significant correlation was found between the PCS and RCS scores (*r* = − 0.10, *p* = 0.53) (Online Resource 1). Hence, these two scores are independent and should be analyzed separately.

### Factors relating to PCS and RCS

**Fig. 4:**
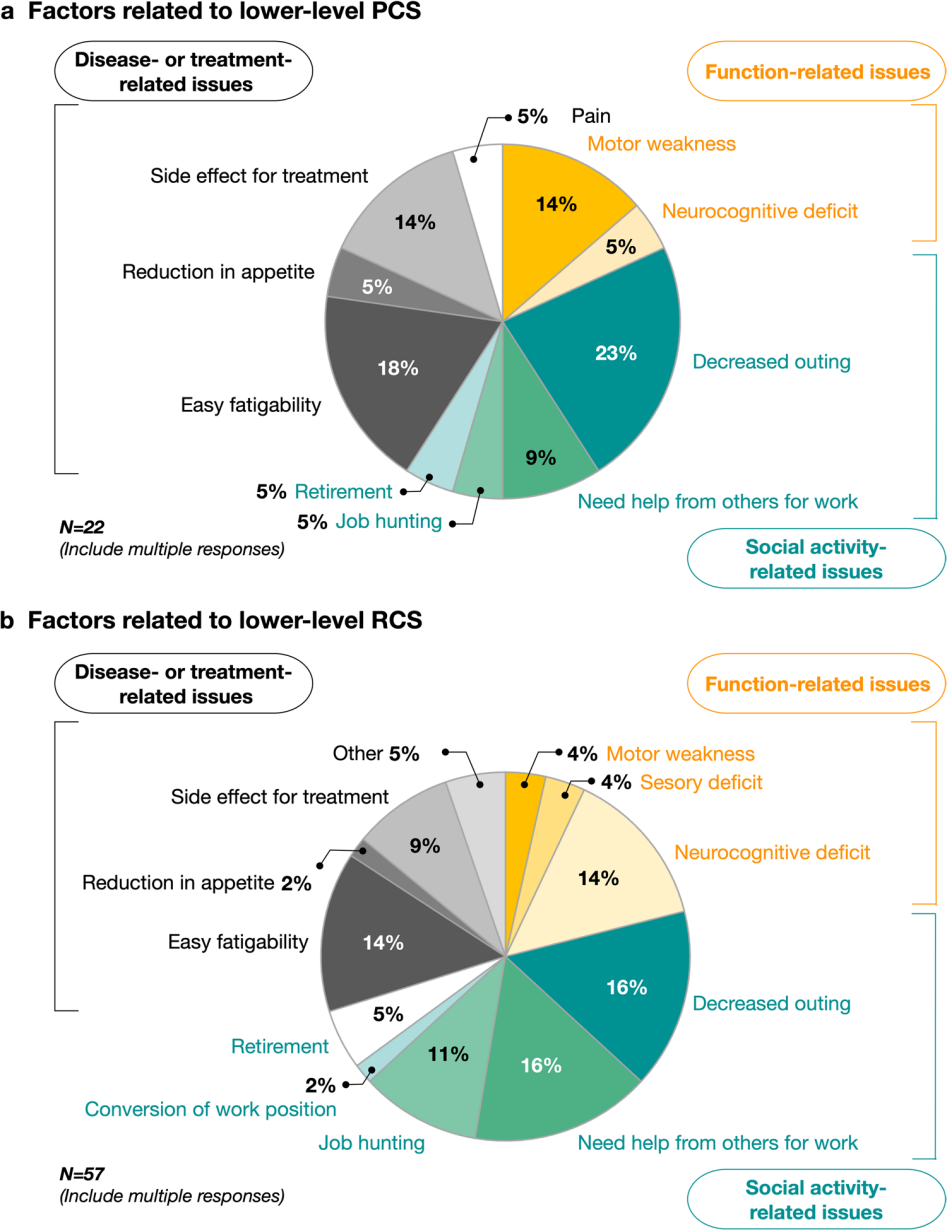
Chief complaints of patients with lower physical component summary (PCS) or role and social component summary (RCS) scores (including multiple responses). The upper and lower columns show the (**a**) PCS and (**b**) RCS scores, respectively. The chief complaints were divided into three categories: function-related issues, social activity-related issues, and disease- or treatment-related issues

The chief complaints (based on multiple responses) of patients with low PCS or RCS scores (< 40) were divided into three categories: function, social activity, and disease- or treatment-related issues. With regard to the PCS score, the major issues included motor weakness (14%), easy fatigability (18%), and decreased outings (23%), which were related to physical activities (Fig. 4a). The main factors causing the decline in the RCS scores were social activity-related issues, including decreased frequency of outings (16%) and the need for assistance with work (16%). Additionally, patients with low RCS scores reported neurocognitive deficits (14%), easy fatigability (14%), and challenges in job hunting (11%) (Fig. 4b).

Univariate analyses were performed to analyze the factors related to PCS and RCS scores (Table 2). Among these factors, motor function was the only factor related to the PCS scores (*p* = 0.0048, effect size [r] = 0.44). Moreover, processing speed was the only factor related to the RCS scores (*p* = 0.030, *r* = 0.35). To note, there is no significant relationship between processing speed and motor function (*p* = 0.70, *r* = 0.19, Online Resource 2). Subsequently, the multiple regression analysis was performed. To identify the independent variables, a stepwise analysis was carried out using the following items: general cognitive function, processing speed, attention, memory, emotion recognition, social cognition, visuospatial cognition, verbal fluency, language, motor function, sensation, and visual field. For the PCS, motor function was the only factor associated with the PCS score. This variable was used as the independent variable in the regression analysis. Motor function was significantly correlated with the PCS score (*p* = 0.0054, Table 2). Following the same procedure, processing speed was identified as the only factor significantly correlated with the RCS score (*p* = 0.030).

**Table 2:** Related functional factors influencing on summary component score

Factor	PCS		RCS	
	Univariate analysis	Multiple regression analysis with stepwise method	Univariate analysis	Multiple regression analysis with stepwise method
General cognitive function	0.52	-	0.54	-
Processing speed	0.44	-	0.030^*^	0.030^*^
Attention	0.74	-	0.83	-
Memory	0.59	-	0.88	-
Emotion recognition	0.32	-	0.48	-
Social cognition	0.94	-	0.76	-
Visuospatial cognition	0.14	-	0.21	-
Verbal fluency	0.96	-	0.75	-
Language	0.60	-	0.28	-
Motor function	0.0048^*^	0.0054^**^	0.25	-
Sensation	0.54	-	0.72	-
Visual field	0.35	-	0.41	-

Pearson’s correlation analysis or t-test were used as univariate analysis. Minus (-) indicates a factor which was not chosen as a possible explanatory variable. ^*^p < 0.05, ^**^p < 0.01

**Fig. 5:**
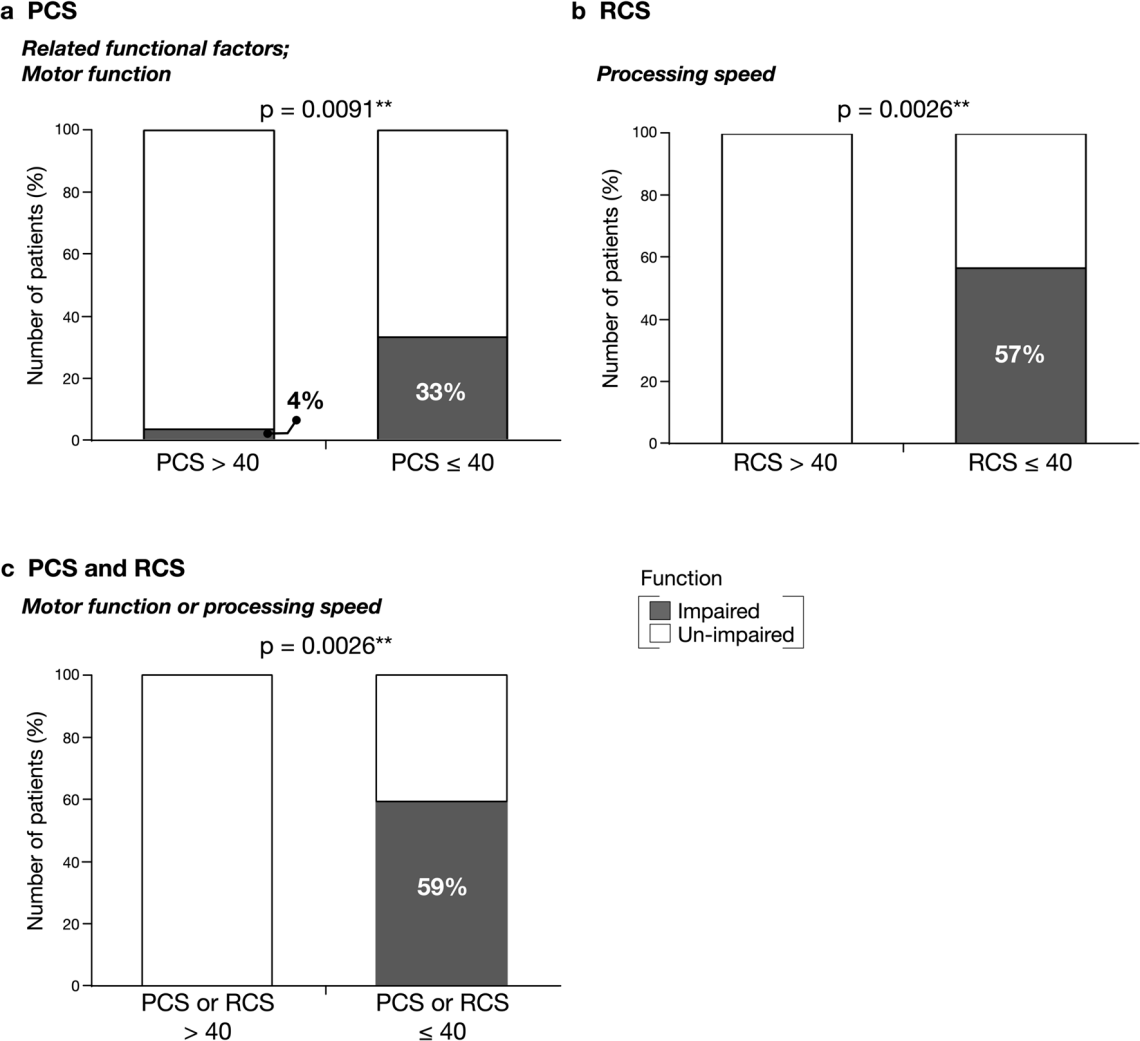
Relationship between brain functions and summary component scores. The brain functions were compared between the low and normal (**a**) physical component summary (PCS) and (**b**) role and social component summary (RCS) groups. The PCS score was associated with motor function, while the RCS score was associated with processing speed. The normality of motor function or processing speed was then compared in patients with low and normal PCS or RCS scores (**c**). Chi-square test; dark color: impaired; light color: un-impaired. ^**^p < 0.01

To confirm the results, the patients were divided into the low-level (≤ 40) and normal groups (> 40), depending on the summary component score, and compared based on the presence or absence of functional impairment using the chi-square test. Motor function was significantly impaired in 33% of patients with low PCS scores (*p* = 0.0091, *r* = 0.41) (Fig. 5a). With regard to the RCS scores, the processing speed was significantly lower in the low-level RCS group (57%) than in the normal RCS group (0%, *p* = 0.0026, *r* = 0.40) (Fig. 5b). Next, the participants were defined as the “impaired group” if either of the two relevant functions, motor function and processing speed, were not normal, or the “un-impaired group” if both were normal. Of the patients with decreased PCS or RCS scores, 59% had impaired motor function or processing speed, while all patients with preserved PCS or RCS scores exhibited normal motor function or processing speed (*p* = 0.0026, *r* = 0.48) (Fig. 5c).

The PCA results demonstrated that neurocognitive functions could be explained in three components (Online Resource 3). The first factor accounted for 37.1% of the variance explained, with factor loadings above 0.4 in all neurocognitive domains except for attention. Therefore, the first factor was considered overall cognitive function. Among these, general cognitive function, processing speed, social cognition, verbal fluency, and emotion recognition, which required rapid information processing abilities, had high factor loadings of more than 0.6 (factor loadings = 0.72, 0.71, 0.68, 0.66, and 0.63, respectively). The second factor, reflecting social communication, included emotion recognition and language (factor loadings = 0.61 and 0.52, respectively) and described 14.3% of the variance explained. The third factor consisted of only one functional domain, including attention (factor loading = 0.77, variance explained = 13.3%). We then compared scores of principal component loadings between the low-level RCS group and the normal RCS group (Online Resource 4). Among these three components, patients with normal RCS scores showed significantly higher scores in component 1, or overall cognitive function (t-test, *p* = 0.018, *r* = 0.38) compared to the low-level RCS group. There were no significant differences in scores of principal component loadings for social communication and attention between the low-level and normal RCS groups (*p* > 0.80).

## Discussion

This study objectively assessed various neurocognitive functions in 40 patients newly diagnosed with GBM who underwent surgical resection to investigate the functional factors influencing QOL. The results showed that the PCS and RCS scores were lower, while the MCS score was preserved in patients with GBM. The factors that influenced the PCS and RCS scores were motor function and processing speed, respectively. All patients with a preserved QOL showed normal motor function and processing speed. Although it has long been well known that QOL in patients with GBM is less favorable than that in healthy people due to several causes [14, 20, 36, 39, 48, 49], this study is the first to demonstrate accurate neurological and neurocognitive functions affecting QOL in GBMs.

Consistent with previous studies [2, 16], the physical aspect of QOL in our patient group was significantly lower than that in healthy individuals. The chief complaints of patients with decreased PCS scores included motor weakness, easy fatigability, and the occurrence of treatment-related side effects, which are the primary factors associated with poor QOL [3, 6, 31]. Interestingly, decreased outings were the most commonly reported concerns among patients with poor PCS. It is presumed that tumor- or surgery-related neurological deficits, easy fatigability, and treatment-related side effects lead to a decreased frequency of outings, resulting in decreased physical and subjective well-being.

Previously, the social implications of GBM received little attention. Previous studies investigating the return to work among patients with GBM have shown that only a low proportion (20–30%) of patients who had some work before surgery could return to work [18, 42]. Considering that GBM typically occurs at an older age, many patients have already retired from work at the time of onset. However, supporting patients in fulfilling their societal and familial roles is crucial for preserving their subjective well-being [44]. In this study, 75% of patients obtained low RCS scores. Clinicians involved in the treatment of GBM should consider these important findings. Following the completion of initial treatment, planning treatments that help preserve functional abilities, enabling patients with GBM to fulfill their societal and familial roles, is crucial for maintaining QOL.

As expected, motor weakness was the only factor associated with the PCS scores. Consistent with our results, previous studies revealed that neurological deficits, including motor weakness, lead to a low QOL and decreased survival [31, 34, 46]. New postoperative neurological deficits may reduce QOL and even decrease the survival benefit of various therapies [46]. Therefore, maintaining motor function should be considered when devising treatment strategies, including surgery. Interestingly, processing speed was associated with the RCS score. A previous study examining the relationship between neurocognitive functions and QOL in patients with temporal GBMs reported a significant correlation between processing speed and social well-being [32]. Processing speed is related to most neurocognitive domains, including general cognitive function, attention, long-term memory, working memory, and fluency [28, 47]. Impairment in processing speed can lead to simultaneous deficits in various neurocognitive functions. Therefore, a decline in processing speed may prevent the smooth interchange of social interactions [41, 43]. For the above reasons, rather than the declining processing speed itself, the reduction of various neurocognitive functions caused by a decrease in processing speed may be a cause of low RCS. Processing speed is a neurocognitive function that tends to decline in patients with GBM [17]. Thus, processing speed may be an important postoperative treatment target among the neurocognitive functions. Other functional domains, except processing speed, were not associated with the RCS score in our patient group. In GBM, the factors influencing the RCS may include general symptoms, which are governed by a broad area of the brain, rather than local symptoms. In lower-grade gliomas, RCS scores are influenced by executive function, which is not a local function [31].

Only a few studies have evaluated the relationship between brain function and QOL in GBM using objective measures. A previous study investigated the relationship between several neurocognitive functions with objective assessments and QOL, revealing that memory, executive function, and processing speed showed direct correlations with health-related QOL in GBM patients [32]. However, these assessments were conducted on pre-operative patients. Another study with 26 glioma patients demonstrated that aphasia severity impacted QOL, but it included both high and low grades of gliomas and did not focus on GBMs [16]. Considering all these facts, our study, which examines the direct relationship between neurocognitive functions and QOL in post-operative GBM patients, is valuable and indicates the need for further research in this area.

This study has some limitations. First, this study focused on the functional factors that influenced QOL at 6 months after surgery. However, other factors, such as older age, sex, tumor location, mood disorder, optimistic thinking, frequency of social contact, treatment such as chemotherapy, and the extent of resection, may also influence QOL [14, 20, 36, 39, 48, 49]. Accurately assessing QOL in patients with GBM remains challenging as QOL is a subjective assessment of patient well-being. Moreover, completing self-reported questionnaires can be difficult for patients with GBM due to language deficits, neurocognitive dysfunction, and easy fatigability [9, 20]. Some patients were excluded from this study because of severe neurocognitive decline or their condition. This problem has long been identified, and several attempts have been made to overcome this issue. In patients with GBM, the KPS is occasionally utilized as a proxy for QOL, as it reflects the level of independence in daily life [9]. In certain instances, a proxy may complete the questionnaires on behalf of the patients [19, 22]. These alternative methods are beneficial for patients who are unable to independently complete the QOL assessment; however, they may not always accurately reflect the patient’s actual QOL [9, 31]. Unfortunately, an optimal solution to this challenge has yet to be identified. A simple and sensitive method for estimating the QOL must be developed, even in patients with a neurocognitive decline. In this study, a lower-level QOL was observed in all patients with impaired motor function or processing speed. Therefore, the QOL of patients with GBMs can be estimated by assessing neurocognitive functions, including motor function and processing speed, which are easier to assess than the QOL. Moreover, the neurocognitive domains investigated in this study were limited as these assessments were performed as part of standard care. Including additional cognitive domains could yield further insights. Another matter with neurocognitive tests used in this type of research is that scores of tests do not necessarily reflect one functional domain, but may sometimes be influenced by other functional domains. Further validation studies using larger sample sizes and QOL assessments applicable to a greater number of patients with GBMs are needed.

## Conclusions

In patients with GBM, the PCS and RCS scores were lower. The PCS score is linked to motor function, while the RCS score is associated with processing speed, both of which are considered key functions. Notably, all patients with impaired motor function or processing speed in the chronic phase also exhibited decreased QOL. Accordingly, maintaining motor function and processing speeds at levels comparable to those of healthy individuals of the same age could indicate QOL maintenance in patients with GBM. We believe our results are effective for all clinicians to make therapeutic strategies including surgery and post-surgical treatments in GBMs. Furthermore, these results may help clinicians in treating GBMs by estimating the QOL of patients, especially in cases where assessing QOL is difficult due to neurocognitive decline or the patient’s condition.

## Supplementary Information

Below is the link to the electronic supplementary material.

Online Resource 1 Correlation between physical component summary (PCS) score and role and social component summary (RCS) score. Pearson’s correlation analysis. (PNG 126 kb)

High resolution image (TIFF 222 kb)

Online Resource 2 Relationship between motor function and processing speed. We compared the score of processing speed between the impaired and not impaired motor functions group and found no significant difference between them (p=0.070, T-test). Red line, mean; blue line, standard deviation. (PNG 145 kb)

High resolution image (TIFF 245 kb)

Online resource 3 (DOCX 16 kb)

Online Resource 4 Relationship between principal component loading of each component and the RCS score. As for component 1 (a), for overall cognitive function, patients with normal RCS scores > 40 showed significantly higher scores compared to the low-level RCS group (scores ≤ 40). There are no significant differences between the normal RCS group (RCS > 40) and the low-level RCS group (scores ≤ 40) in Component 2 (b), the social communication function, and Component 3 (c), the attention function. T-test; red line, mean; blue line, standard deviation. (PNG 285 kb)

High resolution image (TIFF 652 kb)

## Abbreviations

GBMs: glioblastomas
KPS: Karnofsky Performance Status
MCS: mental component summary
MMSE: Mini-Mental State Examination
PCS: physical component summary
QOL: quality of life
RCS: role, and social component summary
SF-36: 36-Item Short Form Survey
