# Supplementary figures and images for: Postsurgical motor function and processing speed as predictors of quality of life in patients with chronic-phase glioblastoma
Source: Acta Neurochir (Wien). 2024 Aug 31;166(1):357. doi: 10.1007/s00701-024-06245-1 (PMC11365834; doi:10.1007/s00701-024-06245-1)

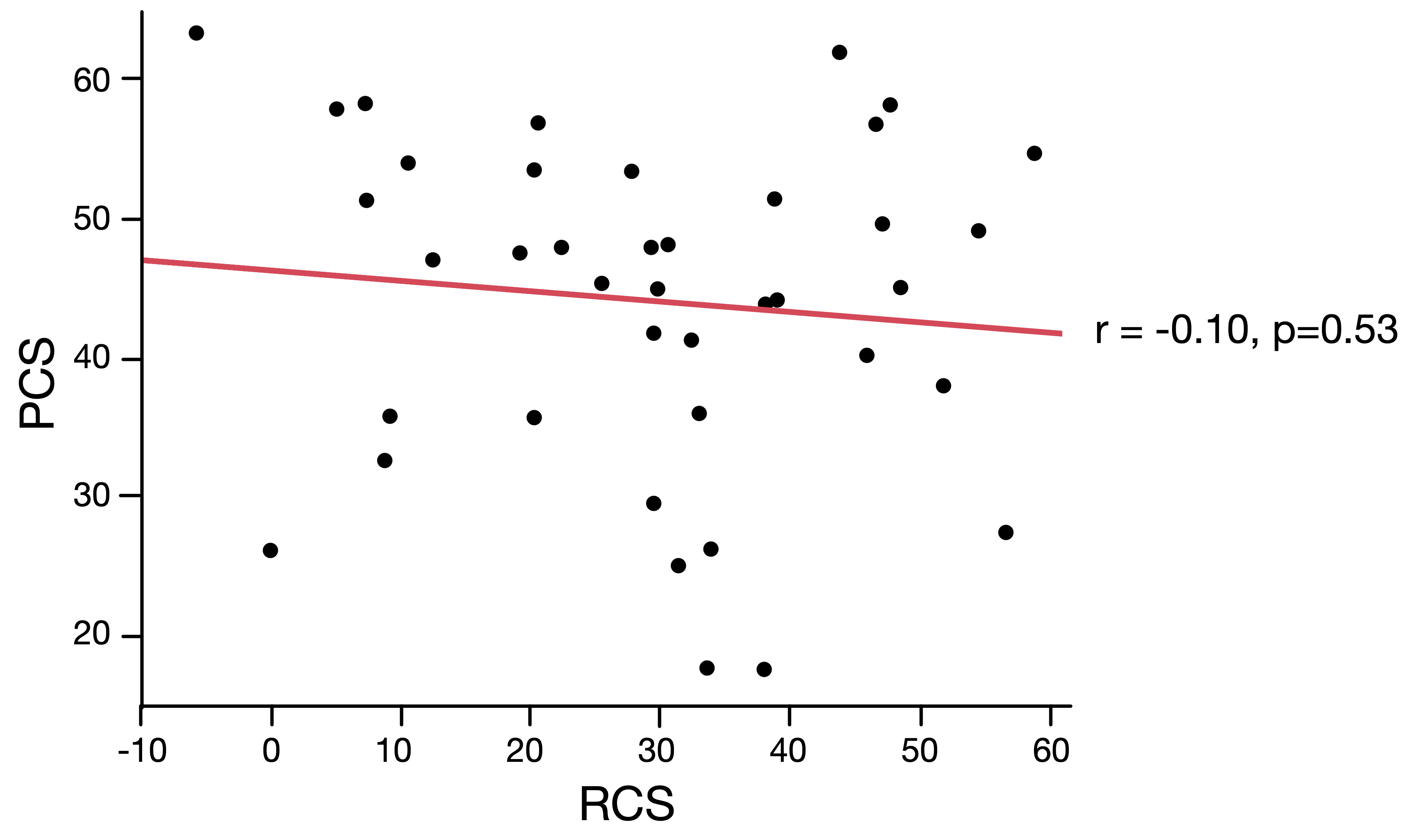

Supplement: Supplementary file 1 — Correlation between physical component summary (PCS) score and role and social component summary (RCS) score. Pearson’s correlation analysis. (PNG 126 kb) [file 701_2024_6245_Fig6_ESM.png]

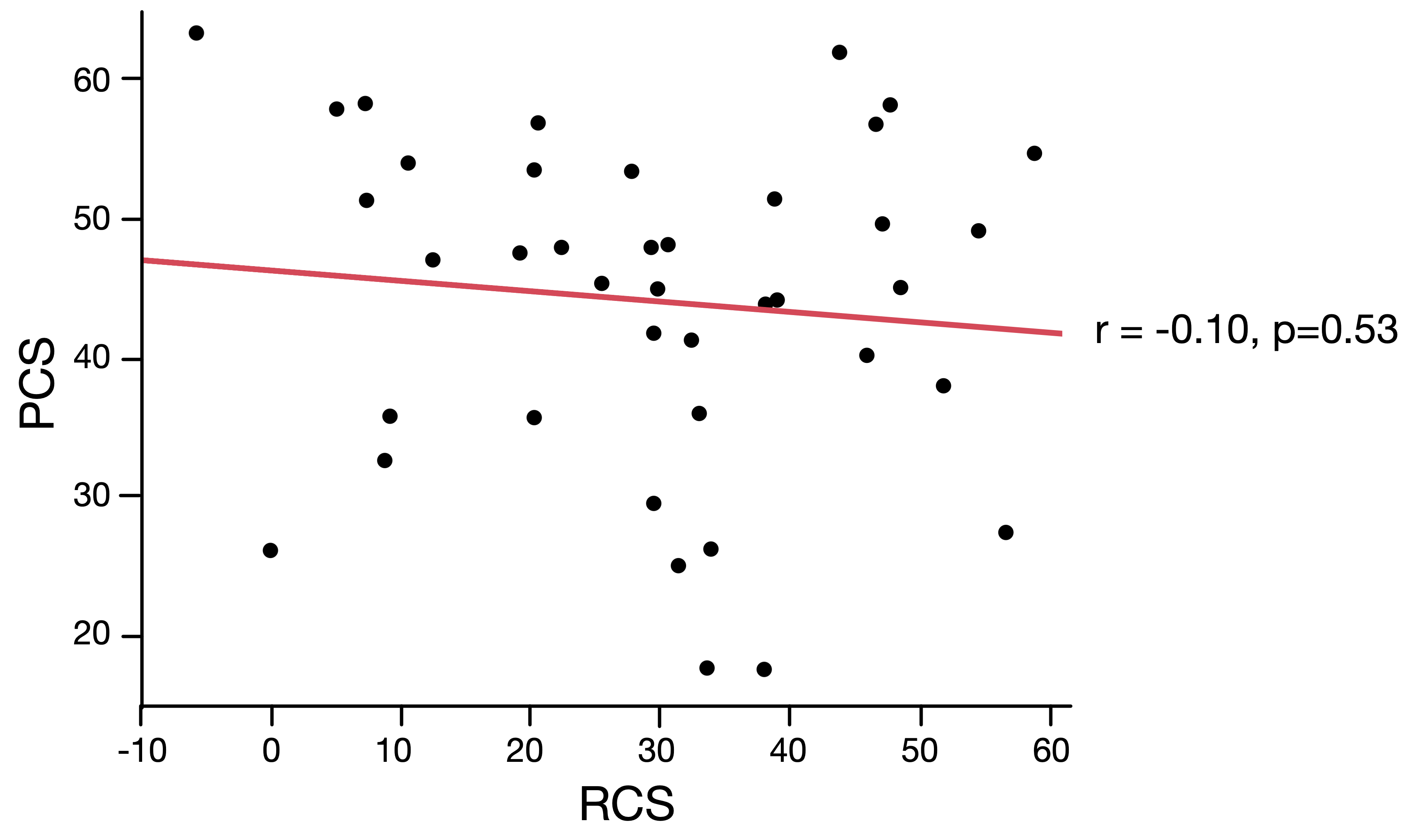

Supplement: Supplementary file 2 — High resolution image (TIFF 222 kb) [file 701_2024_6245_MOESM2_ESM.tiff]

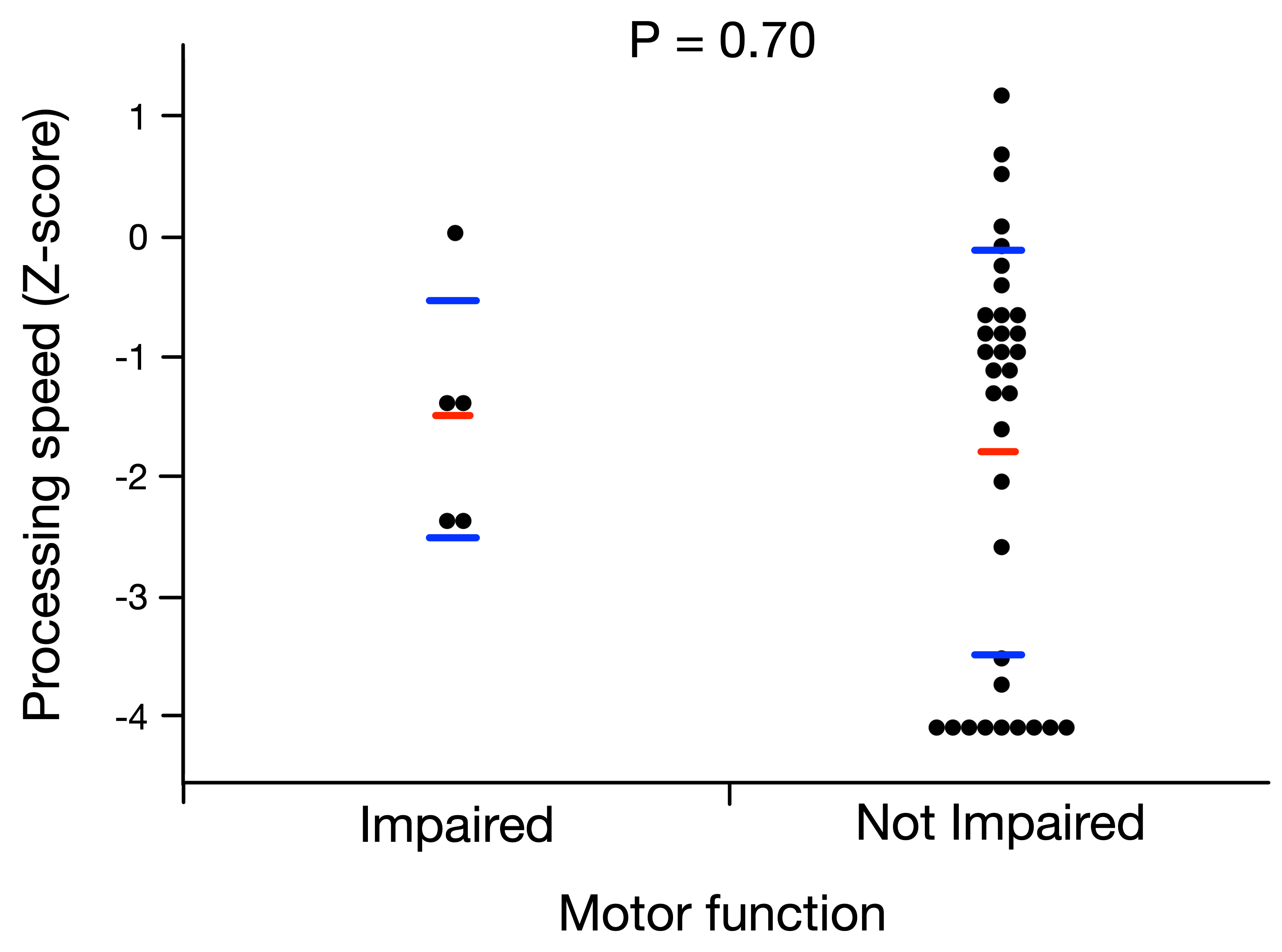

Supplement: Supplementary file 3 — Relationship between motor function and processing speed. We compared the score of processing speed between the impaired and not impaired motor functions group and found no significant difference between them (p=0.070, T-test). Red line, mean; blue line, standard deviation. (PNG 145 kb) [file 701_2024_6245_Fig7_ESM.png]

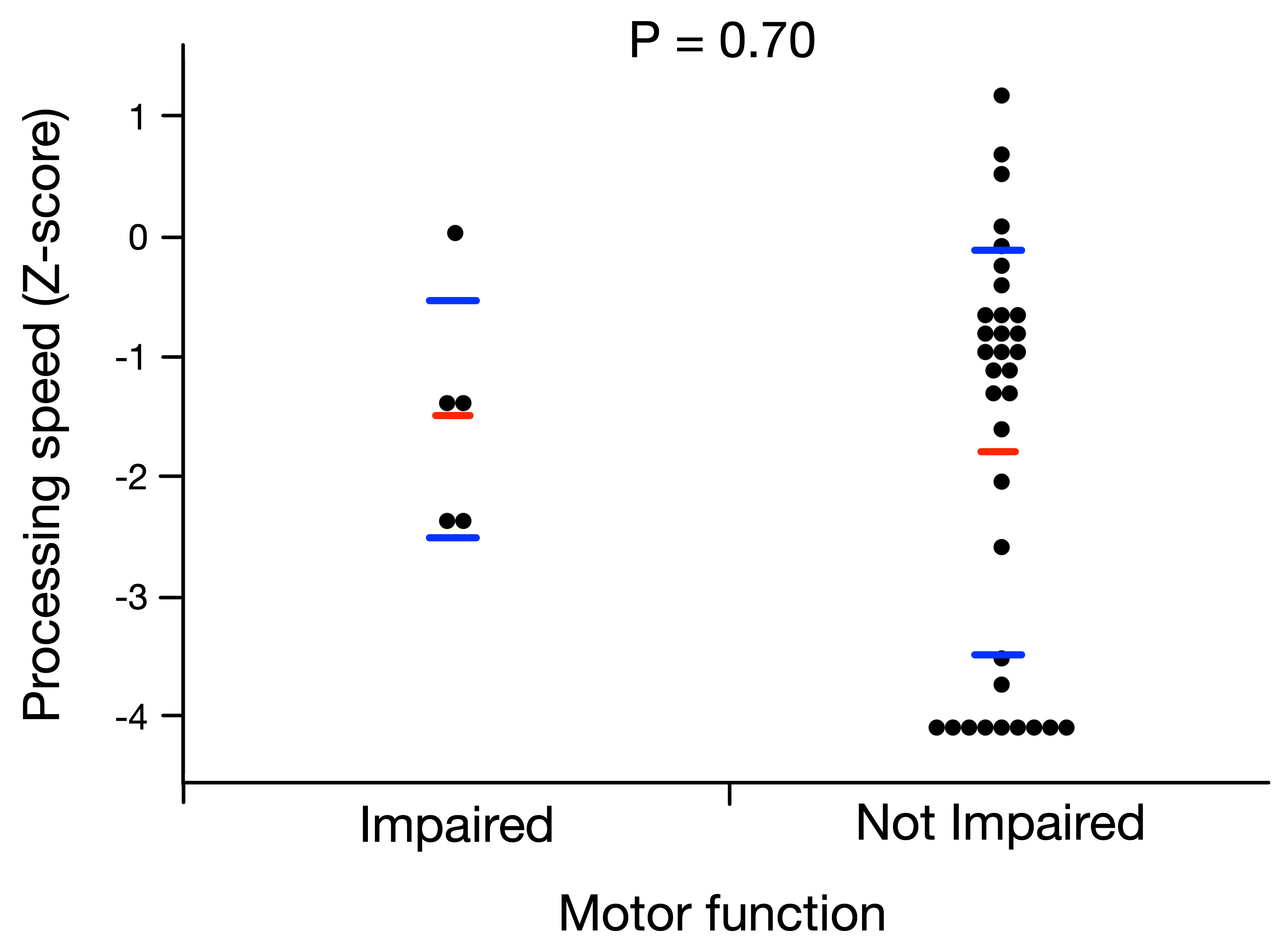

Supplement: Supplementary file 4 — High resolution image (TIFF 245 kb) [file 701_2024_6245_MOESM4_ESM.tiff]

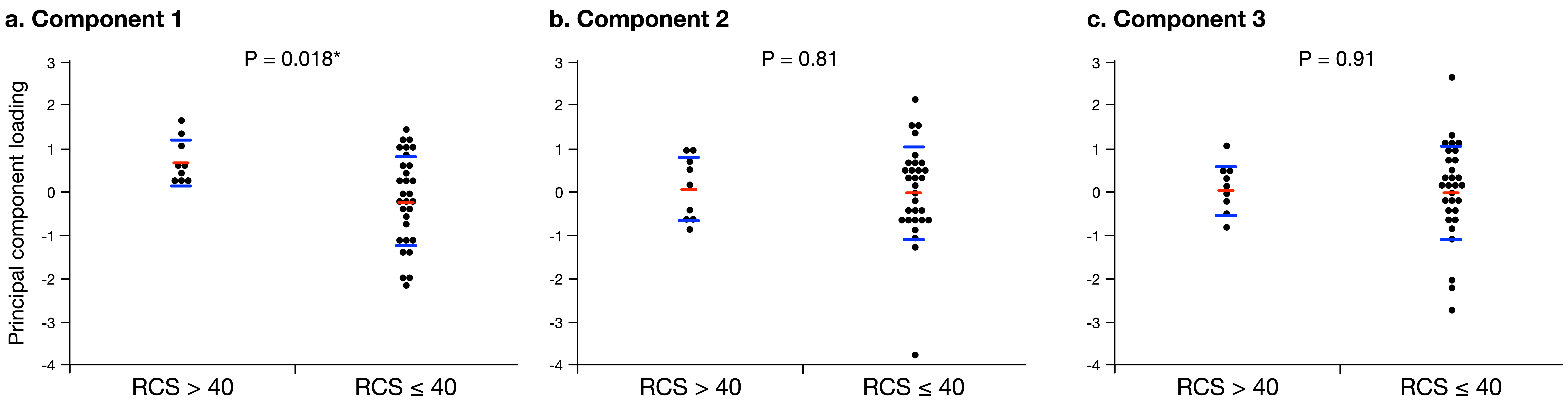

Supplement: Supplementary file 6 — Relationship between principal component loading of each component and the RCS score. As for component 1 (a), for overall cognitive function, patients with normal RCS scores > 40 showed significantly higher scores compared to the low-level RCS group (scores ≤ 40). There are no significant differences between the normal RCS group (RCS > 40) and the low-level RCS group (scores ≤ 40) in Component 2 (b), the social communication function, and Component 3 (c), the attention function. T-test; red line, mean; blue line, standard deviation. (PNG 285 kb) [file 701_2024_6245_Fig8_ESM.png]

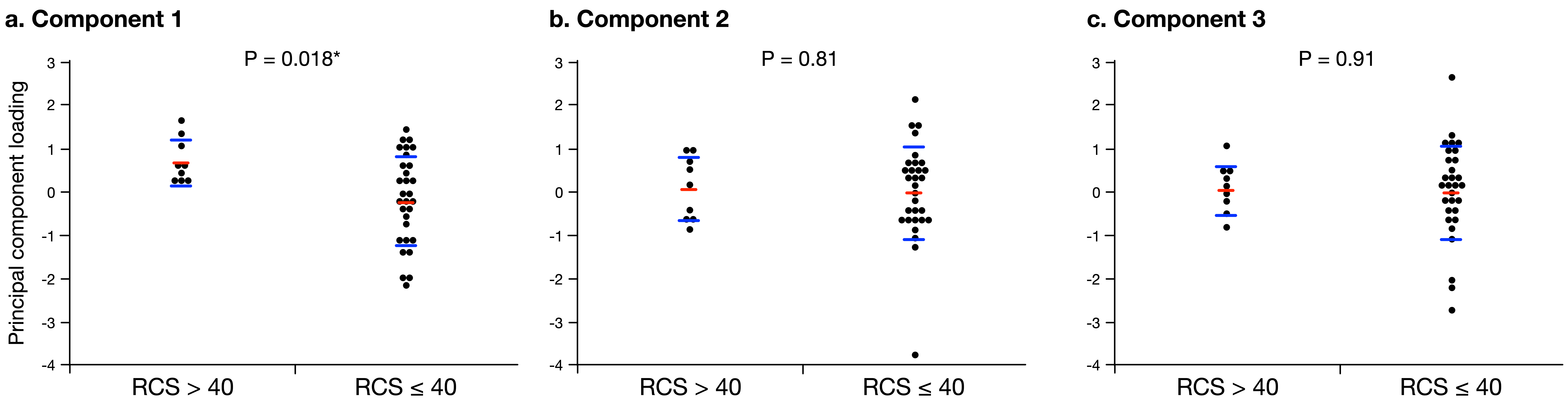

Supplement: Supplementary file 7 — High resolution image (TIFF 652 kb) [file 701_2024_6245_MOESM7_ESM.tiff]
