# Supplementary material for: Postsurgical motor function and processing speed as predictors of quality of life in patients with chronic-phase glioblastoma
Source: Acta Neurochir (Wien). 2024 Aug 31;166(1):357. doi: 10.1007/s00701-024-06245-1 (PMC11365834; doi:10.1007/s00701-024-06245-1)
Supplement: Supplementary file 5 — (DOCX 16 kb) [file 701_2024_6245_MOESM5_ESM.docx]

**Online Resource 3**. Principal component analysis for neurocognitive function

|  | **Component 1** | **Component 2** | **Component 3** |
| --- | --- | --- | --- |
| **Variance explained** | 37.1 | 14.3 | 13.3 |
| **Eigenvalues** | 3.3 | 1.3 | 1.2 |
| General cognitive function | **0.72** | -0.26 | -0.34 |
| Processing speed | **0.71** | 0.13 | -0.12 |
| Social cognition | **0.68** | 0.19 | 0.24 |
| Verbal fluency | **0.66** | -0.41 | -0.23 |
| Emotion recognition | **0.63** | **0.61** | 0.17 |
| Memory | **0.58** | -0.26 | -0.086 |
| Visuospatial cognition | **0.57** | -0.53 | 0.37 |
| Language | **0.50** | **0.52** | -0.45 |
| Attention | 0.35 | 0.11 | **0.77** |

Bold, Significant factors based on the amount of variance explained for components with eigenvalues above 0.40.
